# Role of Endothelin in the Induction of Cardiac Hypertrophy *In Vitro*

**DOI:** 10.1371/journal.pone.0043179

**Published:** 2012-08-17

**Authors:** Tepmanas Bupha-Intr, Kaylan M. Haizlip, Paul M. L. Janssen

**Affiliations:** Department of Physiology and Cell Biology and D. Davis Heart Lung Research Institute, College of Medicine, The Ohio State University, Columbus, Ohio, United States of America; Temple University, United States of America

## Abstract

Endothelin (ET-1) is a peptide hormone mediating a wide variety of biological processes and is associated with development of cardiac dysfunction. Generally, ET-1 is regarded as a molecular marker released only in correlation with the observation of a hypertrophic response or in conjunction with other hypertrophic stress. Although the cardiac hypertrophic effect of ET-1 is demonstrated, inotropic properties of cardiac muscle during chronic ET-1-induced hypertrophy remain largely unclear. Through the use of a novel *in vitro* multicellular culture system, changes in contractile force and kinetics of rabbit cardiac trabeculae in response to 1 nM ET-1 for 24 hours can be observed. Compared to the initial force at t = 0 hours, ET-1 treated muscles showed a ∼2.5 fold increase in developed force after 24 hours without any effect on time to peak contraction or time to 90% relaxation. ET-1 increased muscle diameter by 12.5±3.2% from the initial size, due to increased cell width compared to non-ET-1 treated muscles. Using specific signaling antagonists, inhibition of NCX, CaMKII, MAPKK, and IP3 could attenuate the effect of ET-1 on increased developed force. However, among these inhibitions only IP3 receptor blocker could not prevent the increase muscle size by ET-1. Interestingly, though calcineurin-NFAT inhibition could not suppress the effect of ET-1 on force development, it did prevent muscle hypertrophy. These findings suggest that ET-1 provokes both inotropic and hypertrophic activations on myocardium in which both activations share the same signaling pathway through MAPK and CaMKII in associated with NCX activity.

## Introduction

Cardiac hypertrophy is a form of myocyte remodeling that can be induced by both physiological and pathological stresses. Numerous studies have highlighted the effects of pressure overload and endogenous substances on the hypertrophic response of the heart. Among these substances, endothelin has been of interest for well over a decade, due to the association in stretch-induced inotropic and hypertrophic responses [1], [2]. However, its mechanism of action remains incompletely understood. Endothelin exists natively in three subtypes (ET-1, ET-2, and ET-3) with ET-1 produced in endothelium and myocytes. Endothelin-1 is a potent vasoconstricting agent and within the heart functions mainly as a positive inotrope, chronotrope, and stimulator of the renin-angiotensin-aldosterone system [3].

Inotropic and hypertrophic effects of ET-1 have been widely investigated on cardiomyocytes [4], [5], [6], [7], [8]. The mechanism of action of ET-1 on G-protein coupled receptors mainly activates phospholipase C which hydrolyzes phosphatidylinositol 4,5-biphosphate to diacylglycerol and inositol 1,4,5-trisphosphate (IP3) [9]. IP3 then activates an increased in intracellular Ca^2+^ levels, while diacylglycerol causes the translocation of protein kinase C (PKC) resulting in activation of the small G-protein Ras and consequently, the extracellular signal regulated kinase 1/2 (ERK1/2) cascade [10]. Along with the effects on the hypertrophic response, these messengers could also mediate the intracellular Ca^2+^ transients and myofilament Ca^2+^ sensitivity, subsequently, affecting contractility [9], [11], [12]. It however still remains unclear whether there is one specific signaling cascade or more than one that orchestrates the modulation of inotropic activity and induction of cardiomyocyte hypertrophy.

In the present work, we demonstrate the effects of ET-1 on inotropic and hypertrophic responses using cultured rabbit trabeculae in the absence of systemic regulation and preload. Previous studies from our lab have shown the feasibility to induce hypertrophy via culturing muscles *ex vivo* at high preloads [13], [14]. We use this system to further elucidate the mechanism of the ET-1 induced hypertrophic response and alterations in the inotropic response, with the working hypothesis that ERK1/2 activation is a major contributor to both responses. While the mechanism of action of ET-1 on cardiac hypertrophy still remains elusive, we were able to show that 1) the addition of ET-1 during the culture of intact muscle preparations in the absence of preload leads to an increase in the size and force production of that muscle over time, indicating a hypertrophic response, 2) Na^+^-Ca^2+^ exchanger, CaMKII, and MAPK are involved in both inotropic and hypertrophic effects of ET-1, and 3) there is only a weak association between ET-1 induced inotropic and hypertrophic response and ERK 1/2 activation.

## Materials and Methods

The present study conforms to the NIH Guide for the care and Use of Laboratory Animals (NIH publication No.85-23, revised 1996). All of the animals handled and experiments conducted according to a protocol (2009A0174) approved by the review board of the animal care and use committee of The Ohio State University.

### Multicellular Myocardial Culture

The cardiac trabeculae culture procedure has been detailed previously. Our lab and those of others have shown that these cultured multicellular preparations muscles remain stable in their protein expression/generation [15] and contractile function for up to 5 days [16]. Multicellular preparations can be used from various species [14], [17], including human [16], and this system allows for functional protein product expression of virus-mediated gene transfer [18], [19] and the observation of slow process such as load-induced changes in protein expression [13], [20] or apoptosis [21], [22]. Briefly, New Zealand White rabbits (1.5–2.0 kg) were heparinized and anesthetized by infusion of pentobarbital sodium (50 mg/kg) into the ear vein. After foot-pinch and eye-touch reflexes were absent, hearts were rapidly excised and retrogradely perfused in a modified Langendorff perfusion system with a BDM-containing low calcium Krebs-Henseleit solution. Non-branched, linear trabeculae from the free wall of the right ventricle were dissected and then mounted between a force transducer and a micromanipulator screw a semi-closed circuit culture system. The solution was exchanged with a normal Krebs-Henseleit solution (1.5 mM Ca^2+^) and the muscles were stimulated at 1 Hz at 37°C as previously described [14]. The muscles were then subjected to isometric contractions by stretching to a low passive tension of around 1 mN/mm^2^. Force and kinetics of contraction were continuously monitored for 25 hours. Endothelin-1 was added into the medium to a concentration of 1 nM at 60 minutes after contractions were initiated, whereas inhibitors or 0.1% DMSO (solvent control) were added 30 minutes prior to endothelin administration.

### Pharmacological Antagonists

Several pharmacological agonists and antagonists were used to evaluate the signaling mechanism of endothelin-induced cardiac hypertrophy. For the Ca^2+^-dependent hypertrophic pathway, KB-R7943 (0.5 µM) and KN-93 (1.0 µM) were applied for the inhibition of reverse-mode Na^2+^-Ca^2+^ exchange and CAMKII, respectively [23], [24]. Cyclosporine A (1.0 µM) was used to attenuate calcineurin activity [25], while INCA-6 (40 µM) prevented NFAT-calcineurin association [26]. GF109203X (3.0 µM) is a non-specific PKC inhibitor [27], and 2-APB (4.0 µM) is an IP3 receptor blocker [28]. For the MAPK pathway, PD98095 (10 µM) was utilized in order to inhibit MAPKK activation [29]. An Akt inhibitor (10 µM) was used to attenuate possible Akt-dependent cardiac hypertrophy [30]. Based on previous report that phosphodiesterase type 5 inhibition could attenuate cardiac hypertrophy through cGMP degradation, T-0156 (0.1 µM), a potent phosphodiesterase type-5 inhibitor was applied [31]. Concentrations of these compounds used were at 10 to 100 times their K_i_ value. Since a single experiment takes up to 3 full days to set-up and collect and an additional 3–6 days to analyze, a dose-response curve for each inhibitor used would take 4–5 months per drug to conduct, and was deemed beyond the current capability and scope.

### Protein Electrophoresis of ERK1/2 Phosphorylation

Using only the middle 2/3 of the trabeculae (to prevent including tissue from potentially damaged-ends), Immunoblot-analysis was performed using a standard protocol [14]. Anti-phosphorylated ERK 1/2 (1∶1000) and anti-total ERK (1∶2000) antibodies were obtained from Cell Signaling Technology. By randomized distribution of all samples over multiple gels, blot-to-blot variation impact on statistical analysis was minimized.

### Data analysis and Statistics

Force and kinetics of contraction were recorded and calculated off-line using custom designed (LabView-based) program. Immunoblot densitometry was calculated using the ImageJ 1.37 v program (NIH). Multiple group comparisons were performed using ANOVA followed by Bonferroni post-hoc analysis. Muscle hypertrophy was determined by a comparison of muscle diameters before and after 25 hours of culture using Student paired T-test as described previously [14]. Values are given as mean ± SEM. A two-tailed P value of <0.05 was considered to be statistically significant.

## Results

### Endothelin Affects Myocardial Contractile Performance and Hyperotrophy

**Figure 1:**
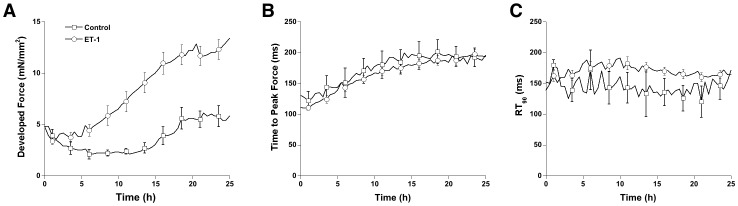
Cultured cardiac trabeculae continuously electrically stimulated to twitch contract at 1 Hz for 25 hours at a preload on only 1 mN/mm^2^ in presence (ET-1, n = 17) and absence (Control, n = 14) of 1 nM ET-1. Compared to control, in ET-1-treated muscles an enhanced active developed force was demonstrated (A), whereas time to peak force (B) and 90% of relaxation time (RT_90_, C) were similar between two groups.

To evaluate whether ET-1 could induce a hypertrophic response, and/or functional change in contractile function in mammalian myocardium, cardiac trabeculae under low preload, were subjected to isometric contractions at 1-Hz electrical stimulation (37°C, pH 7.4) in the presence or absence of 1 nM ET-1 for up to 24 hours. Muscle diameters were compared before and after experiments to assess overall cardiac hypertrophy. We found that ET-1 induced a gradual increase in developed force throughout the 24-hour culture period (Figure 1A). Developed forces were significantly higher in ET-1 treated muscles than in control muscles (no ET-1) after three hours of incubation with ET-1. After 24 hours, ET-1 treated muscles showed a 4.7±0.6 (P<0.05) fold increase in developed force (based on average individual change, overall the group-averaged increase was ∼2.5 fold), while developed force increased only slightly (1.6±0.3 fold, P<0.05) in the control group. No change in time to peak force or in time to 90% relaxation was detected in either control muscles or ET-1 treated muscles (Figure 1B and C). Since the various contractile timing kinetics are linked [32], [33], we did not analyze additional contractile parameters.

**Figure 2:**
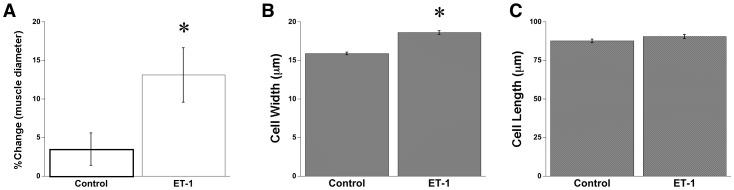
Endothelin-1-induced cardiac hypertrophy. A: % changes in muscle diameter showed a significant increase in ET-1 treated muscle (n = 11), but not in control group (n = 5) by pair’s t-test. Significantly increase in myocyte width (B) but not in myocyte length (C) confirmed hypertrophic effect of ET-1. Values are mean ± SEM from 3 muscles in each group. *P<0.05 vs. control by student t-test.

Hypertrophic effects of ET-1 were revealed by a significant increase in muscle diameter and cell width (Figure 2). While control muscles demonstrated a minimal (3.5±2.1%) increase in muscle diameter compared to their initial size, the presence of ET-1 significantly increased diameter by 12.5±3.2% (P<0.05). To confirm the hypertrophic effect of endothelin-1 at the myocyte level, and to rule out potential edema, cultured cardiac trabeculae were fixed and myocytes were isolated as previously described [14]. Compared to controls, myocytes from ET-1 treated muscles showed an increase in cell width by 16.8% compared to controls (Figure 2B). No significant difference in the length of myocytes between the two groups was observed (Figure 2C). This result indicates that increased muscle diameter by ET-1 was a result of myocyte hypertrophy, and not tissue edema. Hence, this first series of experiments demonstrated that ET-1 directly exerts inotropic effects on the myocardium in which gradual increases in force could be at least partially due to increased number of functional sarcomeres added in parallel in cardiomyocytes.

### Endothelin Induces Cardiac Inotropy

**Figure 3:**
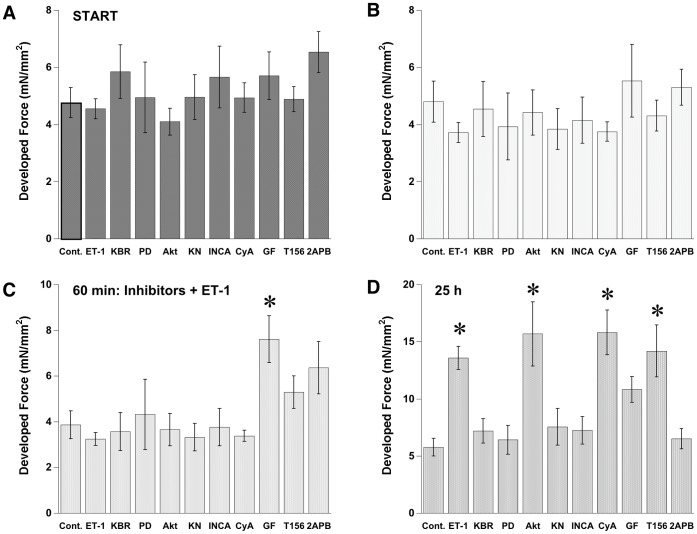
Effect of signaling inhibitors on developed force before and after ET-1 treatment. No significant difference in developed force at start among group as well as 30 min later before inhibitors was added (A, B). After 30 min incubation with GF109203X, significant increase in developed force was observed (C). Significant increases in developed force by ET-1 after 24 hours treatment was observed by the inhibitors of NCX, MEK-1, CaMKII, NFAT-calcineurin association, PKC, and IP3 receptors (D). Values are mean ± SEM from 5–8 muscles in each group. *P<0.05 vs. control by ANOVA.

**Figure 4:**
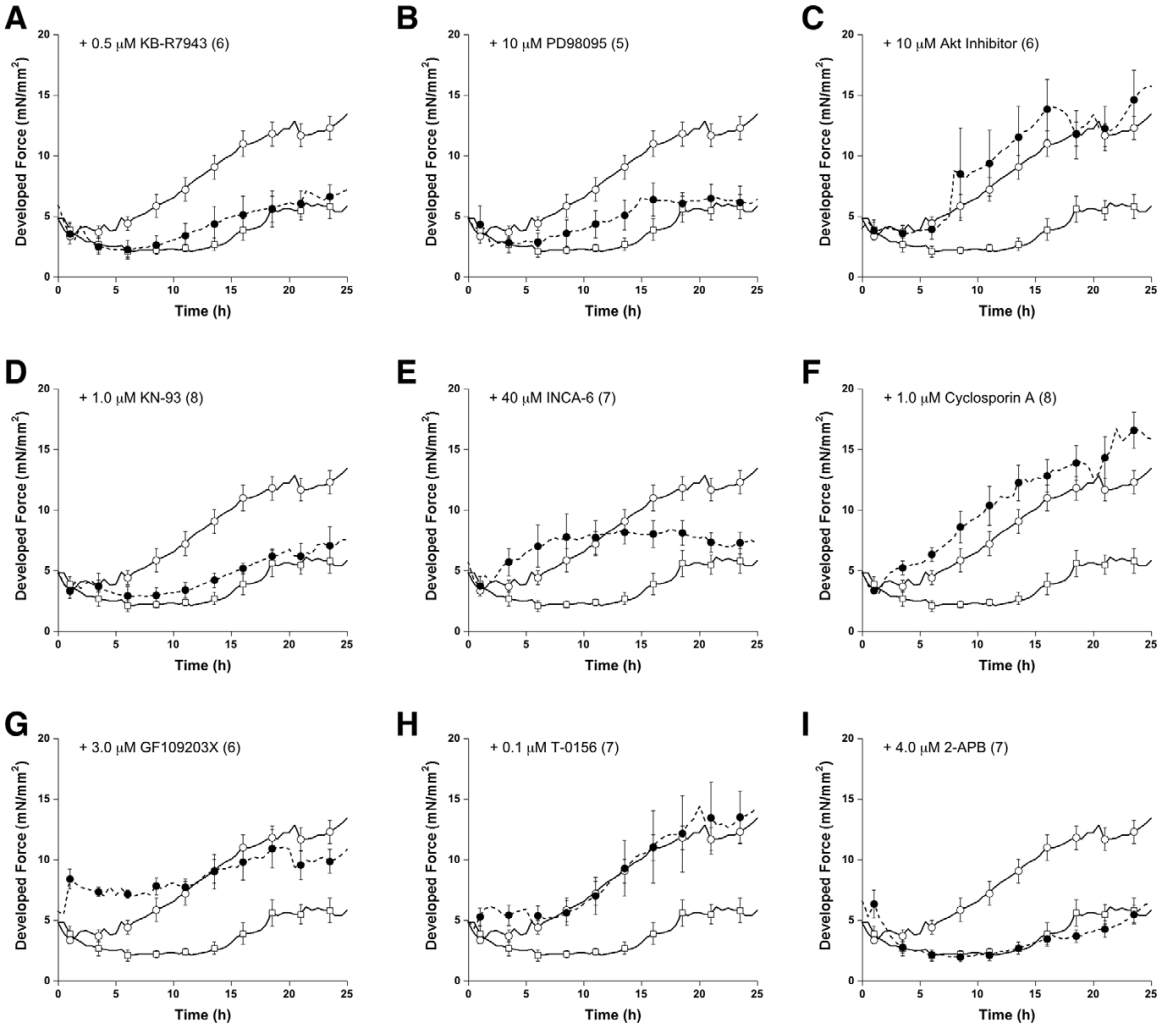
Temporal resolution during 24 hours of impact of signaling inhibitors on developed force. Values are mean ± SEM from 5–8 muscles. The control muscles and the ET-1 treated muscles are included for each inhibitor for direct comparison. Concentration of inhibitor (all in presence of ET-1) and number of experiments are indicated in each panel. Data was analyzed in one ANOVA test.

It has been argued that Ca^2+^-signal-dependent cardiac hypertrophy is due to increased diastolic Ca^2+^ level or altered Ca^2+^ cycling [34], [35]. To test whether an ET-1 induced long-term inotropic response is associated with enhanced Ca^2+^ cycling or enhanced MAPK signaling pathway, various signaling inhibitors were applied to the cultured cardiac trabeculae. Within half an hour before ET-1 was applied, no significant effect of inhibitors on developed force was observed except in the muscle treated with 3.0 µM GF109203X, a non-specific PKC inhibitor (Figure 3B). Figure 3D shows the response to inhibitor after 24 hours. At 25 hours in culture; KB-R7943, PD98095, KN-93, INCA6, and 2-APB inhibited the ET-1-induced increase in force of contraction. Although no significant difference in developed force between ET-1 treated muscles and ET-1+ GF109203X treated muscles was observed after 24 hours, slight increases in developed force from the beginning indicated that the PKC inhibitor potentially attenuates the inotropic effect of ET-1 (Figure 4G). Interestingly, the presence of the inhibitor of NFAT-calcineurin association (INCA) inhibited ET-1 induced increases in developed force in a biphasic fashion. Prior to 10 hours incubation with ET-1 there was no increase in developed force. After 10 hours incubation with 40 µM INCA-6 there was a significant decline in developed force potentially due to a delayed response to downstream inhibition (Figure 4E). The use of cyclosporin A (a direct calcineurin inhibitor) produce a slightly decreased developed force with no blunting of the ET-1 inotropic effect (Figure 4F). This result likely implies that there exists two mechanistic phases underlying an increase in developed force after ET-1 stimulation. In contrast, cyclosporine A, which is proposed to inhibit calcineurin activation [25], could not prevent the inotropic effect of ET-1 suggesting an enhanced response to further downstream targeting of the calcineurin pathway. As expected, Akt inhibition and PDE-5 inhibition did not interfere with the inotropic effect of ET-1.

### Endothelin Signals Induce Cardiac Hypertrophy

**Figure 5:**
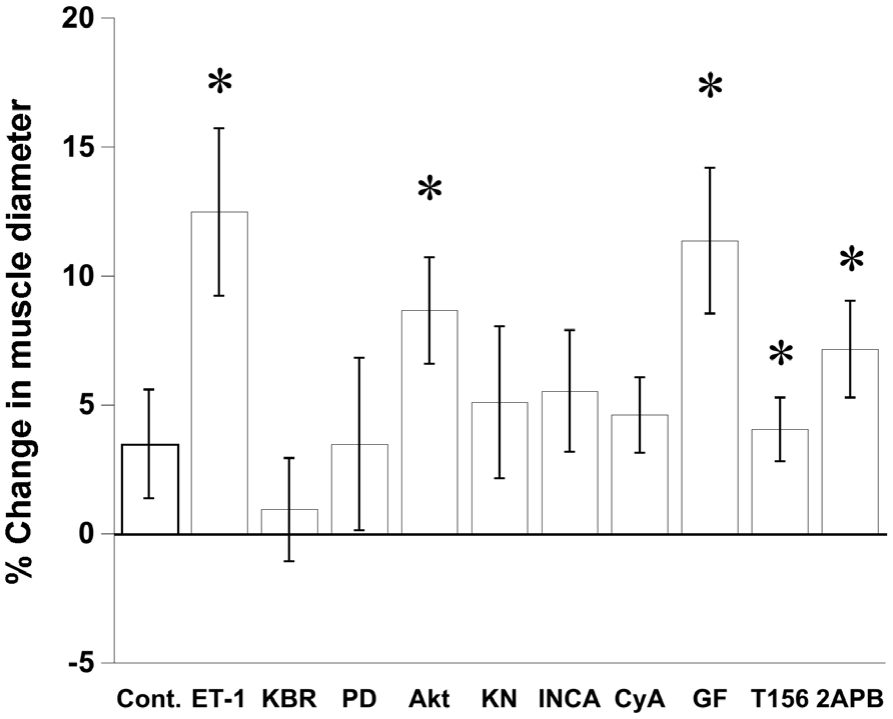
Potential signaling mechanism of ET-1 induced muscle hypertrophy. Muscle diameter before and after culture was analyzed the percent change. Hypertrophy effect of ET-1 was inhibited by the inhibitors of NCX, MEK-1, CaMKII, NFAT-calcineurin association, and calcineurin. Values are mean ± SEM from 5–8 muscles. *P<0.05 compared between before and after culture using paired t-test.

Increases in the diameter of a muscle after 24 hours in culture are a direct parameter determining cardiac hypertrophy (Figure 1). To determine the signaling pathway involved in ET-1 activated hypertrophic response, muscle diameters were recorded after 24 hours in culture for all treatment groups (Figure 5). There was no change in muscle diameter before and after culture in ET-1 treated plus KB-R7943, PD98095, KN-93, INCA6, or cyclosporine A (paired T-test). In contrast, GF109203X and 2-APB, which inhibit the inotropic effect of ET-1, could not attenuate the hypertrophic component of the response. Also, no effects regarding hypertrophy from Akt inhibition and PDE-5 inhibition on ET-1 induced hypertrophy were observed. Results from each set of experiments reveal a general correlation between increased developed force and muscle hypertrophy (Table 1).

**Table 1:** Effect of specific antagonists on inotropic response, muscle hypertrophy, and ERK1/2 phosphorylation in 24-hr endothelin-1 treated trabeculae.

Specific antagonists	Inotropic effect	Hypertrophy	phospho-ERK1/2
KB-R7943*NCX inhibitor*	Inhibit	Inhibit	No
KN-93*CaMKII inhibitor*	Inhibit	Inhibit	No
PD98095*MAPKK inhibitor*	Inhibit	Inhibit	Inhibit
Cyclosporin A*Calcineurin inhibitor*	No	Inhibit	No
INCA6*Inhibiton of NFAT-calcineurin association*	Inhibit	Inhibit	No
2-APB*IP3 receptor blocker*	Inhibit	No	Inhibit
GF109203X*Non-specific PKC inhibitor*	Inhibit *	No	No
Akt inhibitor	No	No	Inhibit
T-0156*Phosphodiesterase type-5 inhibitor*	No	No	No

* GF109203X induced a significant increase in developed force within 1 hour of incubation.

### Endothelin Phosphorylates ERK1/2

**Figure 6:**
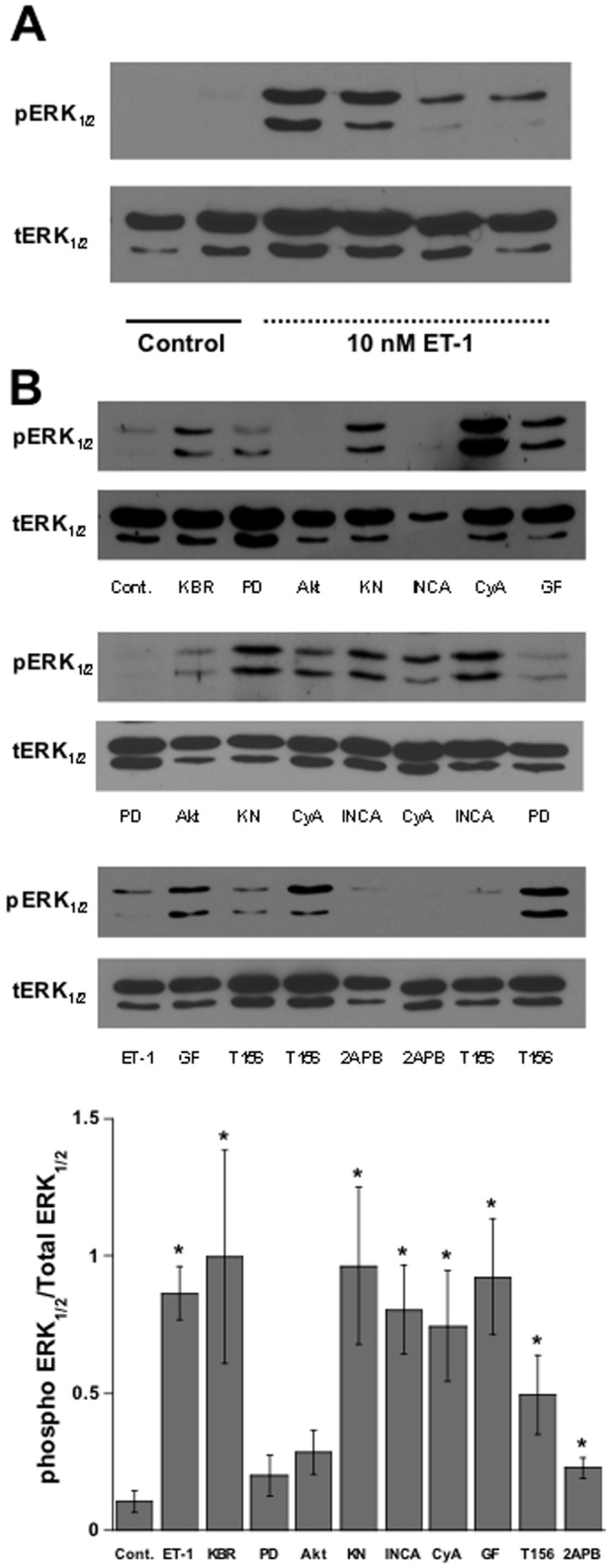
Effect of ET-1 on ERK1/2 phosphorylation. A:Western blot analysis of ERK ½ phosphorylation (pERK) as compared to total ERK ½ (tERK) is increased upon treatment with 1 nM ET-1 in cultured trabeculae. B: Increases in the phospho-ERK1/2 expression in ET-1 treated muscles are augmented by PD98095, Akt inhibitor and 2-APB. Values are mean ± SEM from 4–7 muscles in each group. *P<0.05 vs. control by student t-test.

The MAPK pathway has been established as being involved in the inotropic effects of ET-1 as well as in the induction of cardiac hypertrophy. Previously, it has been suggested that ET-1 stimulated MAPK activation occurred through ERK1/2 signaling [25]. To investigate whether this was occurring in our model, and if it played a role in the hypertrophic response induced by endothelin-1, we first measured phosphorylation of ERK1/2 at 24 hours in culture after ET-1 treatment. We observed that indeed an increase in phosphorylated ERK1/2 levels after exposure with ET-1 occurred (Figure 6A). We then measured the phosphorylation level of ERK1/2, using specific MAPK signaling and calcium signaling antagonists, to determine whether hypertrophy was stimulated through the MAPK pathway. With no clear target of interest, PD98095, a MEK-1 inhibitor, led to the inhibition of the ET-1 induced increase in phospho-ERK1/2. Interestingly, 2APB (an non-specific IP3 inhibitor) and Akt also prevented the ERK1/2 phosphorylation, while the remaining inhibitors tested had no effect. This data, taken in combination with the observed increases in muscle diameter, suggests that ET-1-stimulated cardiac hypertrophy is not specifically related to ERK1/2 activation.

## Discussion

The results of the present study show the effects of endothelin-1 on intact isolated muscle preparations in an *in vitro* culture system using near-physiologically conditions. This study is the first to show an inotropic effect of ET-1, in absence of stretch (preload), on intact muscles developing over time, distinctly different from the acute inotropic effect that is well known and arises in mere minutes [36]. Furthermore, in addition we showed that ET-1 caused an increase in myocyte width, and resulted in an overall increase in muscle mass, indicative of hypertrophy. These observations were made in a multicellular preparation, containing cardiomyocytes, fibroblasts, and endothelial cells. In this system, muscles have the ability to react to biochemical and mechanical stimuli and can remodel and adapt contractile function in a 24–48 hour time span [13], [15], [16], [17], [18], [20], [21], [37]. Thus, results from this model may differ from other *in vitro* models in which isolated cardiomyocytes alone are investigated, or from whole-animal models where systemic regulation is present. Lastly, in an initial attempt to start unraveling the underlying molecular mechanism, we studied potentially involved pathways. Using specific inhibitors, ET-1 activation of the cardiac inotropic response via the activation of MAPK, CAMKII, NCX, and IP3 was determined. In addition, ET-1-induced myocardial hypertrophy is involved in the activation of MAPK, CAMKII, NCX, and calcineurin-NFAT pathway. Although some signaling pathways cross-talk and are involved in both inotropic and hypertrophic responses, we showed for the first time that the effects of ET-1 are independent of each other, yet governed, in part, by similar mechanisms with no obvious dependence on the phosphorylation state of ERK1/2.

Previous studies have shown a correlation between mechanical stress and endothelin release, however, the isolated effects of endothelin on induction of hypertrophy have, to our knowledge, never been observed in a non load-bearing, contracting preparation. There are many endogenous regulators of the hypertrophic response and we have devised a system that allows us to remove these contributors, and look more closely at the mechanism involved with endothelin-induced ventricular hypertrophy. Studies conducted on neonatal cardiomyocytes and rat myocytes have shown that stretch leads to the release of endothelin and hypertrophy [38], [39]. However, the mechanism of action of endothelin release in adult myocardium and in a more physiological model remains unclear. The stretch activated slow inotropic (second phase) response mediated by increased intracellular Ca^2+^ is still being debated as to whether it occurs dependent or independent of ET-1 [40], [41]. Moreover, our previous study showed that neither an ET_A_ receptor antagonist nor a non-selective ET receptor inhibited the increase in developed force after 24-hour of high-load in cultured rabbit trabeculae [13]. Therefore, ET-1 might not be the main signal activating the inotropic response following acute or chronic stretch, and the exact role of ET-1 alone would remain elusive.

Because ET-1 secretion is often correlated with mechanical stretch, the effects of ET-1 alone, in absence of increased mechanical load, would for a large part explain the mechanistic signaling of endothelin on the heart. To determine if ET-1 by itself can produce an inotropic and/or hypertrophic response we studied the effects of ET-1 incubation in absence of changes in stretch. Although our study was not designed to fully elucidate the entire signaling pathway(s) involved, we briefly will discuss a few observations and our interpretation, and will leave the final interpretation of our results to the readership. Based on the specific inhibitors, our results suggest that the ET-1 mediated inotropic response is dependent on NCX, CaMKII, IP_3_, and MAPK activity. Previous studies have determined NCX alone to regulate the stretch induced positive inotropic activity of the heart [40]. Additional studies have revealed that inhibition of MAPK activity accelerated the stretch-induced slow inotropic response and stretch lead to the phosphorylation of p38 and ERK [42]. In the absences of stretch, under ET-1 activation, the inhibition of MAPK depresses the inotropic response. In contrast, it has been suggested that p38 MAPK induces a negative inotropic effect via decreased myofilament Ca^2+^ sensitivity [43]. However, a direct effect of mechanical stretch on calcium related signaling cascades, CaMKII and IP_3_ activation, regarding inotropic activity of the heart has not been established. A previous study shows that direct application of IP_3_ increased the Ca^2+^ spark frequency of isolated ventricular myocyte in which IP_3_ inhibitor could abolish the acute effect of ET-1 on increasing the amplitude of intracellular Ca^2+^ transients [44]. CaMKII inhibition could also attenuate ET-1 increasing cardiac L-type Ca^2+^ current [45]. Therefore, if the mechanical stretch-activated inotropic response occurs mainly through ET-1, both CaMKII and IP_3_ activation should also be involved.

The mechanism underlying ET-1 induced cardiac hypertrophy has been demonstrated for many years; however, studies generally reported only one specific signal at the time. Due to many potential ET-1 signaling cascades being activated, multiple pathways may be responsible for differing effects on the hypertrophic response. Inhibition of NCX, MAPK, CaMKII, and calcineurin-NFAT signals suggested a possible involvement of all these pathways together. Based on the ERK1/2 results, one could speculate that ET-1 might initially activate ERK1/2 phosphorylation. As previously reported, phosphorylated ERK1/2 increased Na^+^-H^+^ exchange activity [46], potential increased intracellular Na^+^ might induce reverse NCX activation [47]. Consequently, increased NCX leads to increased intracellular Ca^2+^ which then activates both CaMKII and calcineurin-NFAT signaling cascades [48]. Between the two signaling pathways, only CaMKII activation can possibly induce an increase in intracellular Ca^2+^ transients [45] and cardiac hypertrophy [49]. However, previous reports on the negative effect of CaMKII on calcineurin dependent NFAT-nuclear translocation [50] suggest that the physiological role of CaMKII likely regulates calcineurin-NFAT signaling activity.

Though the entire complex mechanism still remains unclear, we have furthered our knowledge of potential ET-1 pathways through the use of multiple calcium and MAPK inhibitors, but due to limitations of specificity of antagonists readily available we are currently unable to fully determine the effects of each individual step that eventually lead to hypertrophy. Our data does provide critical novel information in that presence of ET-1 alone (without stretch) leads to an increase in developed force produced in our *in vitro* multicellular model system, where these observations were made in absence of systemic regulation while under controlled mechanical loading conditions. In order to pinpoint the key components in the endothelin-MAPK pathway, future directions would likely need to be focused the effects of inhibition at different points in the pathway. In addition, quantification of the magnitude of responsibility for the different calcium-dependent pathways involved, via dose-dependent test and the inhibition of multiple pathways at a time, would further elucidate the endothelin-induced hypertrophic response.
